# Characterisation of zinc delivery from a nipple shield delivery system using a breastfeeding simulation apparatus

**DOI:** 10.1371/journal.pone.0171624

**Published:** 2017-02-03

**Authors:** Rebekah L. Scheuerle, Sylvaine F. A. Bruggraber, Stephen E. Gerrard, Richard A. Kendall, Catherine Tuleu, Nigel K. H. Slater

**Affiliations:** 1 Department of Chemical Engineering and Biotechnology, BioScience Engineering Research Group, University of Cambridge, New Museums Site, Pembroke Street, Cambridge, United Kingdom; 2 Biomineral Research Group, Medical Research Council Human Nutrition Research, Elsie Widdowson Laboratory, Cambridge, United Kingdom; 3 University College London, School of Pharmacy, Department of Pharmaceutics, London, United Kingdom; Aristotle University of Thessaloniki, GREECE

## Abstract

Zinc delivery from a nipple shield delivery system (NSDS), a novel platform for administering medicines to infants during breastfeeding, was characterised using a breastfeeding simulation apparatus. In this study, human milk at flow rates and pressures physiologically representative of breastfeeding passed through the NSDS loaded with zinc-containing rapidly disintegrating tablets, resulting in release of zinc into the milk. Inductively coupled plasma optical emission spectrometry was used to detect the zinc released, using a method that does not require prior digestion of the samples and that could be applied in other zinc analysis studies in breast milk. Four different types of zinc-containing tablets with equal zinc load but varying excipient compositions were tested in the NSDS *in vitro*. Zinc release measured over 20 minutes ranged from 32–51% of the loaded dose. Total zinc release for sets tablets of the same composition but differing hardness were not significantly different from one another with P = 0.3598 and P = 0.1270 for two tested pairs using unpaired t tests with Welch’s correction. By the same test total zinc release from two sets of tablets having similar hardness but differing composition were also not significantly significant with P = 0.2634. Future zinc tablet composition and formulation optimisation could lead to zinc supplements and therapeutics with faster drug release, which could be administered with the NSDS during breastfeeding. The use of the NSDS to deliver zinc could then lead to treatment and prevention of some of the leading causes of child mortality, including diarrheal disease and pneumonia.

## Introduction

Zinc is an essential micronutrient for paediatric health. It is especially crucial during rapid growth due to its important role in cell division, metabolism, and protein synthesis [1][2][3]. Importantly, it is needed for proper immune system functioning [2].

Zinc deficiency has many consequences including increased risk of infectious disease, stunting, hypogonadism, cognitive impairment, and delayed wound healing [2]. As a result, specifically because of its impact on children under five, it is attributable to 800,000 annual deaths in this age group [2]. In sub-Saharan Africa, 44.3 million children under five are estimated to be zinc deficient [1].

Zinc deficiency is especially prevalent among low-income populations [1]. Plant-based diets are often low in zinc content, and commonly found in resource-limited settings [4]. They are also high in phytates, which decrease the bioavailability of zinc [4].

A study showed that maternal zinc supplementation does not cause extra zinc secretion into human milk, except to a small extent in cases of low zinc content in breast milk of mothers less than 2 months postpartum [5]. Therefore maternal supplementation cannot be used widely to improve zinc delivery to infants [5].

Zinc supplementation has been shown to improve child health by increasing child weight, boosting the immune system, and decreasing the risks associated with lower respiratory tract infections and diarrheal disease [1]. If in just sub-Saharan Africa, 90% of children less than 5 years old received daily zinc supplements, 55 million cases of diarrheal disease, 6 million cases of pneumonia, and 289,0000 deaths could be prevented annually [1].

Zinc supplements in combination with oral rehydration salts have been shown to decrease the duration, and mortality of diarrheal disease [6][1] and are recommended by the World Health Organization [7]. There has been a call by the WHO for studies to further investigate zinc delivery mechanisms to maximise patient compliance and minimise side effects [6]. In response, the nipple shield delivery system (NSDS), a novel method of administering nutrients and medications to breastfeeding infants [8][9][10][11], is being investigated for zinc delivery in the present study.

**Fig 1:**
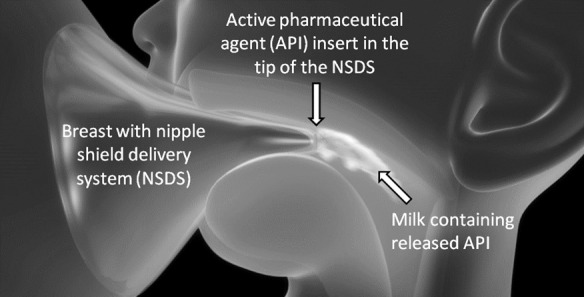
Illustration of the nipple shield delivery system during use. (www.justmilk.org).

The NSDS consists of a silicone nipple shield, adapted to hold a therapeutic-containing (such as zinc) insert. The device is designed so that when it is worn by a mother during breastfeeding, it allows the release of active pharmaceutical ingredient(s) (APIs) into milk consumed by the infant, as illustrated in Fig 1.

The NSDS would be disposable in settings where it would not be possible to ensure the device were cleaned appropriately, such as resource-limited settings lacking potable water. Though zinc supplements are typically administered in water, breast milk can be used as a replacement administration fluid [12][13]. Therefore, the device could be especially useful in resource-limited settings by facilitating zinc delivery via a breast milk suspension.

Using a breastfeeding simulation apparatus, Gerrard et al. have screened model compound release rates from rapidly disintegrating tablet formulations under physiologically relevant breastfeeding conditions [9]. Specifically, release of the model API, sulforhodamine B, was characterised for physiologically relevant ranges of 1–8 mL/min for the milk flow rate and 30–120 pulses/min for the infant suction pulse rate [9].

In the present study, release of a water soluble API, zinc sulfate, was investigated from multiple rapidly disintegrating therapeutic tablet formulations. This was characterised using the breastfeeding simulation apparatus developed by Gerrard et al. [9] to determine feasibility of the NSDS for zinc supplementation. This simulation apparatus serves as the only *in vitro* apparatus for simulating maternal lactation and infant suction in the world, to the best of the authors’ knowledge. Through this study, the impact of tablet composition and manufacturing conditions on API release from the NSDS was investigated.

The zinc content of the tablets was chosen to fall within a range of clinically tested zinc treatment values [2]. Zinc sulphate was used because of its high water solubility, and because it has been shown to have an acceptable taste [4]. It is also included in the WHO list of essential medicines [7].

## Materials and methods

### Human milk

Human milk was donated and mixed from 2 healthy mothers who had given written consent for their milk to be used for research (6.57 ± 0.33 v/v% creamatocrit, 39.4 ± 1.8 g/L lipid, 16.3 ± 1.1 g/L protein, density of 1.02g/mL). All donors were screened negative for HIV 1 and 2, HTLV I and II, Hepatitis B and C, as well as Syphilis. The use of the milk for the purposes of this study received ethical approval from the University of Cambridge’s Cambridge Human Biology Research Ethics Committee. Milk was stored at -80°C, then thawed at 3°C followed by room temperature before use in the study.

Creamatocrit and protein content of the milk was experimentally determined. To measure the creamatocrit, samples were drawn into capillary tubes, then sealed with plasticine before centrifuging in a Sigma 1–14 microcentrifuge with a microcentrifuge rotor (Sciquip, Newtown, UK) at 12000 g for 15 min [14]. The cream layer was measured using vernier calipers in triplicate. Fat content was calculated using Wang et al.’s creamatocrit to fat correlation for thawed samples stored at -20°C [15], assumed to adequately assess the fat content of these samples [9]. Protein content of the milk was measured using a standard Bradford Agent assay (Sigma Aldrich, Dorset, UK) [16].

Milk density was measured using a hydrometer (VWR, Lutterworth, UK) at temperatures in the range produced during the simulation apparatus tests (34.4–34.7°C) which simulate mouth temperatures that could be present during NSDS use [17].

The composition of milk tested fell within the ranges reported in literature [15][14], and the density measured was very similar to the 1.03 g/mL reported previously [18].

### Tablets

Biconvex directly compressed zinc-containing tablets were manufactured in-house based on two compositions using the materials outlined in Table 1. The excipients were chosen based on formulations for rapidly disintegrating tablets in the literature [19]. To formulate the tablets, the API and all excipients followed by the lubricant were initially blended, then sieved at 500 μm followed by a final blending step. Tablets were manufactured using a Manesty F3 tablet press (Manesty, Liverpool, UK) with a biconvex 80 single punch and die set (Holland, Nottingham, UK).

**Table 1:** Laboratory-Manufactured Target Tablet Composition.

Chemical	Role	Composition 1 (%w/w)	Composition 2 (% w/w)	Grade	Manufacturer
**Zinc Sulfate Monohydrate**	API	16.64	16.64	Puram p.a.	Sigma-Aldrich, Dorset, UK
**Lactose Monohydrate (SuperTab 14SD)**	Filler	77.36	67.36	Ph. Eur	DFE Pharma, Goch, Germany
**Sodium starch glycolate (Explotab CLV)**	Superdisintegrant	3.00	3.00	Typ (A) Ph. Eur	Mendell GmbH, Volklingen, Germany
**Croscarmellose sodium (Ac-Di-Sol)**	Superdisintegrant	2.00	2.00	Ph. Eur	FMC Biopolymer, Girvan, UK
**Microcrystalline Cellulose (Avicel PH102)**	Binder	0.00	10.00	Ph. Eur	FMC Biopolymer, Girvan, UK
**Magnesium stearate**	Lubricant	1.00	1.00	Technical Grade	Sigma-Aldrich, Dorset, UK

Tablets were of two different compositions, and compressed with varying forces, resulting in four different types of tablet. Tablet length, height, and width were measured using vernier calipers. The crushing force of the tablets oriented diametrically was measured using an Eweka TBH200 hardness tester (Heusenstamm, Germany). This physical characterisation data is shown in Table 2.

**Table 2:** Physical Characteristics of Tablets using United States Pharmacopoeia Methods.

Tablet Name	Type 1 (T1)		Type 2 (T2)		Type 3 (T3)		Type 4 (T4)	
Composition Type	Composition 1		Composition 2		Composition 2		Composition 1	
	Average	n	Average	n	Average	n	Average	n
**Diameter (mm)**	8.081 ± 0.003	10	8.093 ± 0.003	10	8.081 ± 0.005	10	8.092 ± 0.003	10
**Height (mm)**	3.65 ± 0.03	10	4.65 ± 0.03	10	3.76 ± 0.02	10	4.52 ± 0.03	10
**Width (mm)**	5.16 ± 0.01	10	6.031 ± 0.007	10	5.150 ± 0.008	10	5.906 ± 0.007	10
**Weight (mg)**	331 ± 2	10	332.0 ± 0.7	10	335.7 ± 0.5	10	331.3 ± 0.5	10
**Hardness (N)**	73	10	19	10	143	10	18	10
**Friability (%)**	0.3		1.76		0.16		1.94	
**Compression Force (kN)**	23		22		25		21	

n: number of samples; averages listed ± standard error

Tablet disintegration testing was performed by United States Pharmacopeia (USP) methods with a basket rack assembly disintegration apparatus (Copley, Nottingham, UK) [20].

### Breastfeeding simulation

Breastfeeding practices depend on both the mother and child’s behaviours and physiology, as well as the time of day, time within the feed, and infant’s age. Infants breastfeed for nutritive and non-nutritive purposes, with feed volumes and sucking behaviours differing between the two [21][22].

**Fig 2:**
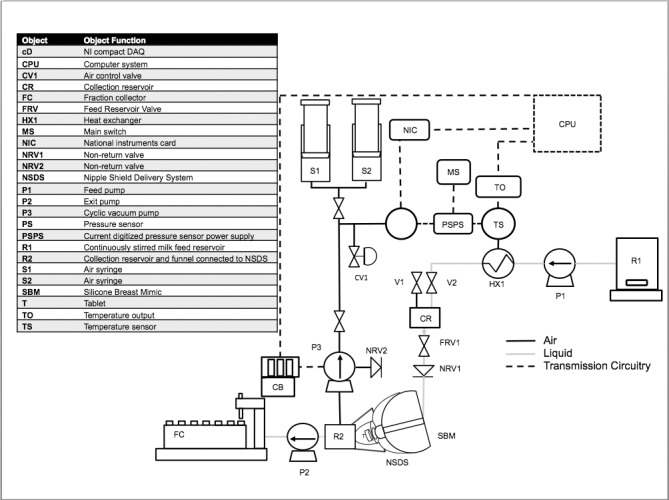
Breastfeeding Simulation Apparatus. (adapted from Gerrard et al., [9]).

The breastfeeding simulation apparatus by Gerrard et al. mimics a mother’s lactation and infant’s suction behaviours during breastfeeding *in vitro* [9]. The apparatus heats human milk to a physiologically relevant temperature range, and pumps it through a silicon human nipple mimic while under suction from a modified breast pump. The flow rates of the milk, suction pressure, and suction frequency are programmable. In the present study, the NSDS loaded with a therapeutic tablet was inserted into this apparatus for zinc release testing under physiologically relevant breastfeeding conditions. The device was positioned after the human nipple mimic as shown in the process flow diagram in Fig 2.

**Fig 3:**
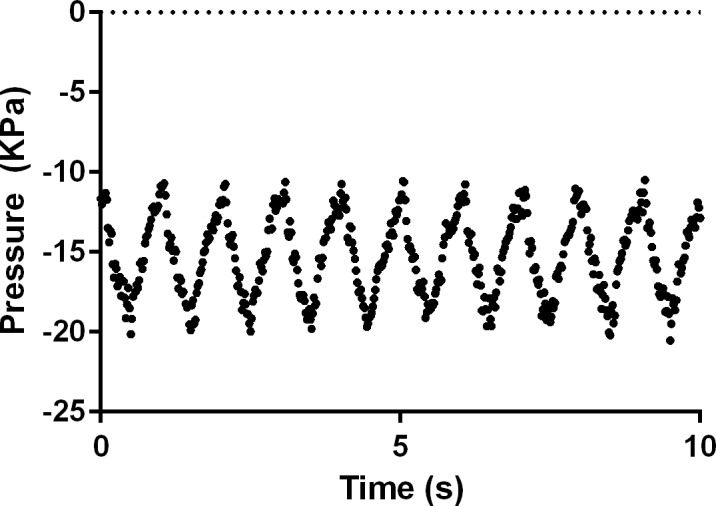
Representative pressure profile in the breastfeeding simulation apparatus. Values are representative of that of a suckling infant, and were measured from the breastfeeding simulation apparatus over 10 seconds occurring several minutes into a trial run testing tablet T2.

The suckling rate of an infant ranges widely, being reported as 46–126 cycles per minute [23] with peak suction reported as -145 ± 58 mmHg (-19.3 ± 7.7 Kpa) and baseline vacuum being reported at -64 ± 45 mmHg (-8.53 ± 6.00 Kpa) [24]. The milk flow rate varies from 0.4–16.8 mL/min [9]. Therefore, a representative suction frequency of 60 suction/min and milk flow rate of approximately 5 mL/min were chosen for this experiment, as tested by Gerrard et al. previously [9]. The instrument was set to a physiologically relevant pressure range and amplitude, with a peak vacuum of -21.5 ± 0.08 Kpa and a baseline vacuum of -9.58 ± 0.07 Kpa. A representative pressure profile is shown in Fig 3.

The experiment was performed in triplicate for each tablet for 20 minutes each, twice the length of time of an average breastfeed [24].

### Detection of zinc concentration by ICP-OES

The zinc concentrations in breast milk samples and in the tablets were determined at the MRC Human Nutrition Research Unit (Cambridge, UK) using an ICP-OES (Jobin Yvon Horiba–ULTIMA 2C) equipped with a concentric glass nebulizer (0-1ml/min sample flow rate), a 50ml glass cyclonic spray chamber and a radial torch with an 3mm i.d. alumina injector. Sample solutions were introduced from an auto-sampler (Jobin Yvon Horiba AS500) using a sample probe with 0.75mm i.d. sample tubing and 0.76mm i.d. pump tubing (black/black). Instrument operating conditions were: 1300W RF power, 12 L/min plasma gas flow, 2 L/min sheath gas flow.

Breast milk samples were prepared by 1:20 dilution volumetrically with diluent composed of ultra-high purity water (Sartorius Stedium Biotech Arium pro UV Water Polisher), 0.002% TritonX100 (Sigma-Aldrich, UK), and 1ppm molybdenum (SPEX CertiPrep Metuchen, NJ) as an internal standard. Samples were then vortexed with a Fisherbrand Whirlimixer (Loughborough, UK).

For the elemental zinc content analysis, tablets were ground using a mortar and pestle and added to ultra-high purity water and incubated at 37°C overnight. The samples were then vortexed and diluted 1:20 volumetrically in diluent before being analysed by ICP-OES.

Calibration standards of zinc (Perkin Elmer, Inc., Shelton, CT) diluted 1:20 volumetrically in diluent with a final concentration of 0.1% nitric acid were prepared to achieve a concentration range of 0.05–50 mg /L.

Zinc and Molybdenum were detected at 213.856 nm and 202.030 nm using a photomultiplier tube voltage of 605V (kg*m^2^*s^-3^*A^-1^) and 955V (kg*m^2^*s^-3^*A^-1^) respectively, with an integration time of 0.5s for both.

Intensities generated from the zinc and molybdenum emission spectra were imported from the ICP Analyst 5.4 Software (Horiba, UK). Two calibration curves, high and low range, were produced for zinc concentrations in the ranges of 0–0.5 mg/L and 0.5–50 mg/L. Zinc concentration in each sample was calculated using the respective linear regression equation from of the ratio of the mean intensity for zinc to the mean intensity for molybdenum. The zinc amount in the original sample was calculated based on the dilution, the sample weight and the milk’s density at the experimental temperature. Zinc concentrations were measured for 9 interspersed samples chosen from 30 samples collected from each breastfeeding simulation. Zinc concentrations of the remaining samples were estimated using linear interpolation. Release profiles based on cumulative zinc release over 20 minutes of running the simulation were then generated. Each experiment was performed in triplicate.

The zinc content of the tablets detected by ICP-OES is shown in Table 3.

**Table 3:** Laboratory-Manufactured Elemental Zinc Content of the Tablets as Measured by ICP-OES.

	T1	T2	T3	T4
Zinc detected by ICP-OES (wt%)*	5.56 ± 0.08	5.37± 0.08	5.41± 0.07	5.40± 0.14

*n = 3, error represents standard error.

Based on Table 3, the elemental zinc content average of the tablets varied from 17.81–18.21 mg depending on the tablet type.

## Results and discussion

Release of zinc from the NSDS requires the API to have either dissolved into the human milk, or to have disintegrated enough into for it to pass through the holes of the NSDS. Zinc release was quantified in breast milk using a novel inductively coupled plasma optical emission spectrometry (ICP-OES) technique, unique because it did not require sample digestion via microwave. This technique is a large step forward in rapid analysis of API release from the NSDS, as milk is inherently a complex fluid in which to detect analytes because of its heterogeneous composition [25]. Other methods for analysing breast milk samples for other analytes have included high-performance liquid chromatography, mass spectrometry, gas chromatography, and high pH anion exchange chromatography, but many of these methods can have limitations due to either low sensitivity, tediousness, high costs, loss of sample, or large sample requirements [25][26].

The total release of zinc that occurred during the breastfeeding simulations from the tablets loaded in the NSDS, as detected via ICP-OES, is summarised in Table 4.

**Table 4:** Tablet Disintegration and Zinc Release Characterisation.

**Tablet Name**	**Type 1 (T1) Composition 1**		**Type 2 (T2) Composition 2**		**Type 3 (T3) Composition 2**		**Type 4 (T4) Composition 1**	
	Average	n	Average	n	Average	n	Average	n
Disintegration time* (s)	137 ± 2	6	136 ± 2	6	147 ± 6	6	132 ± 5	6
Proportion of zinc released	0.51 ± 0.02	3	0.32 ± 0.04	3	0.42 ± 0.04	3	0.42 ± 0.07	3

*Disintegration time measured using USP methods; n: number of samples; averages listed ± standard error.

The disintegration time of the tablets of the same composition and differing hardness was not significantly different (P = 0.3922 for Composition 1 and P = 0.1140 for Composition 2), based on an unpaired t test using Welch’s correction. The disintegration times between T2 and T4, two types of differing composition but with harness differing by just 6%, also were not statistically different by the same test (P = 0.5208).

No tablet type released the full loaded dose of zinc. Ideally the full dose of API would be released from the tablets loaded into the NSDS during use. Total zinc release ranged from 32–51% of the loaded dose for the various tablet types. Although T1 released the most, and T4 the least zinc on average, unpaired t tests using Welch’s correction, indicate that neither the tablets of the same composition, nor those of the same hardness had total zinc release amounts that were statistically different from one another, with (P = 0.3598 for T1 and T4; P = 0.1270 for T2 and T3, and P = 0.2634 for T2 and T4). This test, being based on single values, does not take into account the trend in the data shown in the profiles, which can indicate hardness and compositional effects on zinc release over many time points.

**Fig 4:**
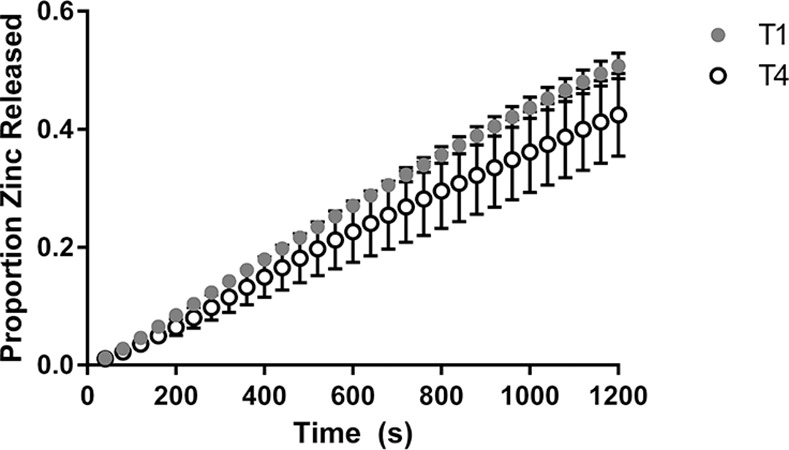
Release of zinc from the NSDS for Tablet Composition 1. Values are based on a weight fraction proportion; n = 3; error bars represent standard error.

**Fig 5:**
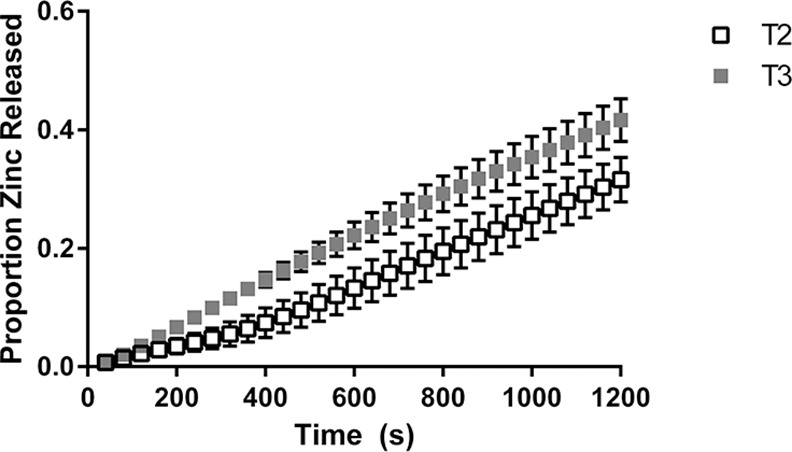
Release of zinc from the NSDS for Tablet Composition 2. Values are based on a weight fraction proportion; n = 3; error bars represent standard error.

Zinc release profiles from the NSDS under physiologically relevant conditions using the breastfeeding simulation apparatus is shown in Fig 4 and Fig 5 for Composition 1 and Composition 2 respectively.

The figures indicate that the average amount of zinc released was higher for the harder tablets of each composition for the duration of the simulations, though as mentioned the total zinc amount released was not statistically different for either pair in Figs 4 or 5.

**Fig 6:**
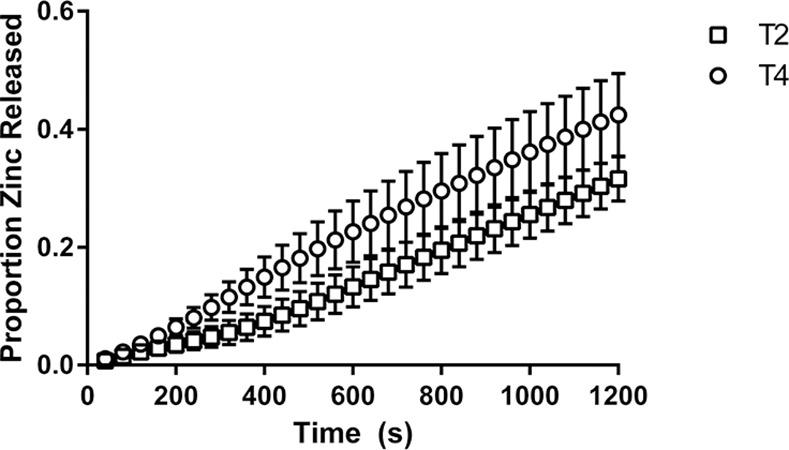
Release of zinc from the NSDS for Tablets of Similar Hardness. Values are based on a weight fraction proportion; n = 3; error bars represent standard error.

By comparing release profiles of zinc for T2 and T4, two types with similar hardness, impact of composition can be seen, as shown in Fig 6.

Fig 6 indicates that the average amount of zinc released over the duration of the simulations was higher for T2 than T4, though the total released amount of zinc was not statistically different as discussed.

To compare the release profiles, *f*_*2*_, the similarity factor as shown in Formula 1, was calculated. This factor, used by the Food and Drug Administration (FDA) and European Medicines Agency (EMA) is typically used in comparing the similarity of dissolution profiles for solid oral dosage forms [27].

$$f_{2}=50*log\;\left[{\left(1+(\frac{1}{n})\sum_{j=1}^{n}{\left(R_{j}-T_{j}\right)}^{2}\right)}^{-.5}x100\right]$$ (1)

The similarity factor is a function of *T*_*j*_, the test value, and *R*_*j*_, the reference value, over all time points, *n*. The factor is 100 when the profiles are identical, and lowers as the profiles become less similar. The FDA and EMA suggest that the profiles can be considered similar when *f*_*2*_ is over 50.

The release profiles of the tablets tested of the same composition were found to be similar to each other (*f*_*2*_ = 63.5 for Composition 1 and *f*_*2*_ = 54.4 for Composition 2). The two tablet types of differing composition and similar hardness also had similar release profiles, with *f*_*2*_ = 53.3.

To potentially deliver higher proportions of the zinc dose, the excipients, surface area, shape, hardness, or size of the zinc tablets tested could be modified. Ultimately dosage amounts, API release rate, and dosage frequency would need to be tailored to the population, need, and pharmacokinetics of the API For example, diarrheal disease treatment of infants less than 6 months requires smaller doses of zinc than for infants older than 6 months [28]. The dosing frequency would be set in accordance with the characteristics of the zinc release profiles of the chosen tablets. Potentially small doses of zinc could be administered regularly throughout the day, rather than one dose per day. This could be advantageous because zinc uptake has been shown to saturate at 5 mg when administered with a meal, but daily zinc uptake can be increased by repeated administration with multiple meals [4]. If used for supplementation rather than diarrheal disease treatment which typically has higher treatment doses, zinc at doses of 2 mg/d of zinc is recommended for infants up to 6 months, or 3 mg/d for children up to 3 years [2]. Therefore tablets with smaller zinc amounts than the tested amounts would be required for use in the NSDS if given as a daily supplement.

Changes in prototype design could facilitate faster API release. Alternatively the dosage amount of the API could also be modified in the supplement and optimised in combination with dosing frequency to ensure appropriate therapeutic and safe dosage of the API to the infant occurs.

The method of using the breastfeeding simulation apparatus combined with the zinc detection method as presented could be used to characterise other zinc-containing tablets. The breastfeeding simulation apparatus could also be used to characterise tablet formulations with alternative APIs, especially those with alternative physicochemical properties. It could also be used to characterise alternative NSDS prototypes in future studies. A modified ICP-OES method could also be used in analysing the API release rate if suitable trace elements are used as alternative APIs.

## Conclusion

Delivery of zinc from tablets using the NSDS was shown as feasible using a breastfeeding simulation apparatus. During the simulations, zinc was released into breast milk pumped through the breastfeeding simulation apparatus under physiologically relevant fluid flow conditions. Inductively coupled plasma optical emission spectrometry was demonstrated as a successful method for detecting the zinc released into the breast milk. This detection technique can be applied to future human nutrition and drug delivery studies.

Administration of zinc from each of four rapidly disintegrating zinc tablet types used in the NSDS was tested. Of the tested tablets, the total zinc release of tablets of differing hardness but the same compositions was not significantly different from one another based on unpaired t tests using Welch’s correction. Further, for tablets of differing composition but similar hardness the total zinc release was not significantly different from another by the same test.

Future work could include testing the efficacy of zinc delivery over a wider range of potential breastfeeding conditions by modifying the tested milk composition, suction rates, and milk flow rates. Alternative tablet compositions, sizes, hardness, or API dosages could also be tested. Furthermore, other dosage forms could be tested with the NSDS. Other NSDS designs could also be investigated to alter the fluid flow through the device and therefore potentially the release properties of the loaded dosage form.

There is a strong need for increased paediatric zinc supplementation and therapeutic availability. If scaled up globally and optimised, the NSDS could potentially be used to administer this nutrient, which could be an influential method of decreasing child mortality, especially due to two of the leading preventable causes of death, pneumonia and diarrheal disease [29].

## Supporting information

S1 File Dataset. Underlying data.
(XLSX)

S2 File Written Permission for Use of Fig 1. (PDF)

## Abbreviations

API: active pharmaceutical ingredient
NSDS: nipple shield delivery system
WHO: World Health Organization
ICP-OES: inductively coupled plasma optical emission spectrometry
USP: United States Pharmacopeia
