# Supplementary material for: Characterisation of zinc delivery from a nipple shield delivery system using a breastfeeding simulation apparatus
Source: PLoS One. 2017 Feb 3;12(2):e0171624. doi: 10.1371/journal.pone.0171624 (PMC5291522; doi:10.1371/journal.pone.0171624)
Supplement: S2 File — (PDF) [file pone.0171624.s002.pdf]

JustMilk

06 January, 2017

Dear Rebekah Scheuerle and other co-authors of the paper “Characterisation of Zinc Delivery from a Nipple Shield Delivery System using a Breastfeeding Simulation Apparatus”,

I hereby grant you permission to use the figure shown below for publication in this paper under the CC-BY 4.0 license. This includes permission to publish the figure in an open access journal, with the understanding that it may therefore be available online to be read, downloaded, copied, distributed, or used in other ways without further permission.

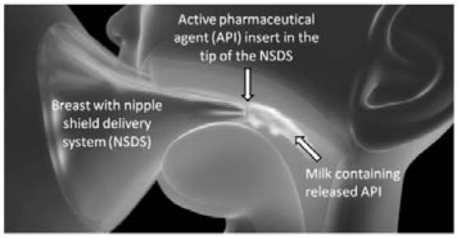

Sincereley,

Dr. Geoff Galgon

CEO JustMilk
